# Gas1 up-regulation is inducible and contributes to cell apoptosis in reactive astrocytes in the substantia nigra of LPS and MPTP models

**DOI:** 10.1186/s12974-016-0643-2

**Published:** 2016-07-08

**Authors:** Xiao-Long Sun, Bei-Yu Chen, Hai-Kang Zhao, Ying-Ying Cheng, Min-Hua Zheng, Li Duan, Wen Jiang, Liang-Wei Chen

**Affiliations:** Institute of Neurosciences, Department of Neurobiology and Collaborative Innovation Center for Brain Science, School of Basic Medicine, Fourth Military Medical University, Xi’an, 710032 China; Department of Neurology, Xijing Hospital, Fourth Military Medical University, Xi’an, China; Department of Orthopedics, Xijing Hospital, Fourth Military Medical University, Xi’an, China; Department of Neurosurgery, Second Affiliated Hospital, Xi’an Medical University, Xi’an, 710038 China; Department of Developmental Biology and Genetics, Fourth Military Medical University, Xi’an, China

**Keywords:** Gas1, Astrocytes, Apoptosis, Substantia nigra, Neuroinflammation, Parkinson’s disease

## Abstract

**Background:**

Reactive astrogliosis is a remarkable pathogenetic hallmark of the brains of Parkinson’s disease (PD) patients, but its progressive fate and regulation mechanisms are poorly understood. In this study, growth arrest specific 1 (Gas1), a tumor growth suppressor oncogene, was identified as a novel modulator of the cell apoptosis of reactive astrocytes in primary culture and the injured substantia nigra.

**Methods:**

Animal models and cell cultures were utilized in the present study. Lipopolysaccharide (LPS)- and 1-methyl-4-phenyl-1,2,3,6-tetrahydropyridine (MPTP)-treated animal models were used to detect Gas1 expression in the brain via immunohistochemistry and western blot. Cell cultures were performed to analyze Gas1 functions in the viability and apoptosis of reactive astrocytes and SH-SY5Y cells by double labeling, CCK-8, LDH, TUNEL, flow cytometry, and siRNA knockdown methods.

**Results:**

Gas1 expressions were significantly elevated in the majority of the reactive astrocytes of the brains with LPS or MPTP insults. In the injured substantia nigras, GFAP-positive astrocytes exhibited higher levels of cleaved caspase-3. In cell culture, the up-regulated Gas1 expression induced apoptosis of reactive astrocytes that were insulted by LPS in combination with interferon-γ and tumor necrosis factor-a. This effect was confirmed through siRNA knockdown of Gas1 gene expression. Finally and interestingly, the potential underlying signaling pathways were evidently related to an increase in the Bax/Bcl-2 ratio, the abundant generation of reactive oxygen species and the activation of cleaved caspase-3.

**Conclusions:**

This study demonstrated that the up-regulation of inducible Gas1 contributed to the apoptosis of reactive astrocytes in the injured nigra. Gas1 signaling may function as a novel regulator of astrogliosis and is thus a potential intervention target for inflammatory events in PD conditions.

**Electronic supplementary material:**

The online version of this article (doi:10.1186/s12974-016-0643-2) contains supplementary material, which is available to authorized users.

## Background

Parkinson’s disease (PD) is one of the most common neurodegenerative diseases in human beings and is characterized by the progressive loss of dopaminergic neurons in the substantia nigra (SN) and axonal terminals in the striatum. Despite previous intensive studies, the mechanisms underlying the degeneration of dopaminergic neurons remain poorly understood. Many studies have demonstrated that neuroinflammation mediated by astrocytes and microglial cells may play a vital role in the death or survival of nigral dopaminergic neurons [1]. Astrocytes, i.e., the most abundant glial cell type in the central nervous system (CNS), can provide essential support for the homeostasis of various CNS neurons, e.g., the supplementation of energy substances and growth factors, the regulation of blood flow, and active participation in synaptic transmission and plasticity [2]. However, in the pathogenesis and progression of PD, astrocytes undergo hypertrophy, proliferation, and dynamic changes in the expression of genes such as glial fibrillary acidic protein (GFAP) and vimentin, and this process is referred to as astrogliosis [3]. Accumulating evidence suggests that these activated astrocytes produce diverse inflammatory factors, such as pro-inflammatory cytokines and nitric oxide (NO) [4, 5], which then contribute to the neurotoxicity of the surrounding dopaminergic neurons. Apparently, the proper regulation of astrocyte-mediated neuroinflammation is essential for the prevention of the progression of PD [6]. One possible method to eliminate the overactivation of astrocytes is the limitation of astrogliosis strength in the inflammatory response. Interestingly, cell apoptosis among the activated astrocytes has been noted in vivo in the brains of Alzheimer’s disease [7] and HIV encephalitis [8] patients. In vitro, astrocytes also undergo apoptosis following strong activation by lipopolysaccharide (LPS) and other inflammatory stimuli [9, 10]. There is a growing consensus that the apoptosis of activated astrocytes may present as a self-regulatory mechanism to control the severity of inflammation in the brain [11]. However, currently, the molecular mechanisms underlying this self-regulation of the apoptosis of astrocytes are still far from clear.

Growth arrest specific 1 (Gas1) is a glycosylphosphatidyl inositol (GPI)-linked protein and a pleiotropic protein found in various cell types. Initially, Gas1 was found to block the cell cycle in the transition from the G_0_ phase to the S phase when a cell culture was deprived of serum or in a confluent state [12]. Subsequently, Gas1 was reported to be capable of inducing apoptosis in different cells, such as gliomas, gastric cancer, and even hippocampal neural cells [13–16]. For mechanical signaling studies, the PI3 kinase and NFkB signaling pathways were proposed to participate in the induction of Gas1 expression in endothelial cells [17]. Additionally, sonic hedgehog signaling is also involved in Gas1 regulation in mammalian embryonic development [18]. Recent studies have confirmed that Gas1 is widely expressed in neurons and astrocytes in the adult CNS [19]. While the engagement of Gas1 in neuronal apoptosis has been observed in SH-SY5Y cells and the hippocampus [15, 16], its potential role in the regulation of astrocytes has yet to be determined. Therefore, in the present study, we focused on the critical role of Gas1 in the self-regulatory apoptosis of activated astrocytes and identified Gas1 as a novel modulator of reactive astrocytes in an animal model of PD and cell culture. The data from this study suggest that Gas1 actively works in cell apoptosis in reactive astrocytes in the manners of increasing reactive oxygen species generation, the Bax/Bcl-2 ratio, and caspase-3 activation during the neuroinflammatory response.

## Methods

### Preparation of the LPS rat model and the MPTP mouse model

Sprague-Dawley rats and *C57BL/6* mice were used in this study and were supplied from the Animal Center of the Fourth Military Medical University (FMMU), China. All animal experiments were performed in accordance with the National Institutes of Health guide for the care and use of Laboratory animals (NIH Publications No. 80-23), revised 1996, and approved by the Committee of Animal Use for Research and Education of FMMU. All efforts were made to minimize animal suffering and reduce the numbers of animals used.

For the preparation of the LPS rat model and the MPTP mouse model, the treatments of the animals were performed as described in our previous studies [20]. Briefly, adult rats received unilateral injections of LPS (Sigma, L6143, 0.5 μl of 10 μg/μl diluted in 0.9 % saline) into the medial forebrain bundle (MFB) at the following coordinates, AP −4.2 mm, L 1.5 mm, and V 7.8 mm, and into the contralateral side with the same volume of 0.9 % saline. Adult mice were administered intraperitoneal injections of MPTP (Sigma, M0896) of 25 mg/kg per day for five continuous days, and the same volume of saline was injected as a control. All the animals were sacrificed at week 1, 2, 3, or 4 after the LPS or MPTP injections. The brain samples were collected for the subsequent immunohistochemistry and western blot experiments.

### Cell cultures of the primary astrocytes and SH-SY5Y cells

The primary astrocytes were isolated from the cortex, striatum, or ventral midbrain of 1–3-day-old neonatal rats as described previously [20]. The dissociated cells were seeded and cultured in 75-cm^2^ flasks coated with poly-l-lysine in Dulbecco’s modified Eagle’s medium-high glucose (DMEM-HG) supplemented with 10 % fetal calf serum and 100 U/ml penicillin/streptomycin in humidified 5 % CO_2_ and 95 % air at 37 °C. When the cells grew to confluence, the flasks were shaken at 280 rpm for approximately 20 h at 37 °C. Immunocytochemistry was performed to guarantee that the percentage of GFAP-positive cells was >95 %. Additionally, the SH-SY5Y cells were also cultured under the same conditions employed for the primary astrocytes.

The astrocytes and SH-SY5Y cells were plated in 60-mm dishes with serum-containing media. After the concentrations reached 80 % confluence, the culture media of the astrocytes were replaced with serum-free media with different LPS (Sigma, L6143) and cytokine (IFNγ 50 ng/ml, TNFα 10 ng/ml) concentrations alone or in combination for the indicated times. For the inducement of Gas1 expression, the media of the SH-SY5Y cells were replaced with serum-free media and cultured for different time periods.

### Transfection of Gas1 siRNA in the cultured primary astrocytes

The knock-down of the Gas1 gene (GenBank accession number NC_005116) expression was performed. For the transfection of the siRNAs, the rat primary astrocytes were seeded in 6-well or 96-well dishes 24 h before transfection with siRNAs targeting Gas1 or control siRNA (Jima) using lipofectamine 2000 (Invitrogen) for 6 h basically according to the manufacturer’s instructions. siRNA duplexes were prepared as follows: sense: 5′-CUACUACGACGAAGAAUAUTT-3′; and antisense: 5′-AUAUUCUUCGUCGUAGUAGTT-3′. At 48 h after siRNA transfection, the astrocytes were treated with LPS, IFNγ, and TNFα for 24 h and then collected for the following western blot or real-time PCR analyses.

### CCK-8 assay and LDH release assay

After the astrocytes were treated with different reagents, the cell viabilities were assessed using the CCK-8 assay according to the manufacturer’s instructions (Nanjing Vazyme Biotech Co.). The lactate dehydrogenase (LDH) release was also assessed to indicate the injury to the astrocytes, using a commercial kit (Beyotime Biotechnology). For the CCK-8 assay, the final data were presented as the percentages of the control levels. For the LDH release measurements, the final data are presented as the percentages of the maximal values.

### Flow cytometry assay

Flow cytometry analyses were also used to quantify the survival, necrosis, and apoptosis of the primary astrocytes. The cultured astrocytes were harvested, and flow cytometry assays were performed via Annexin V-PI staining. The cell survivals, necrosis, early apoptosis, and later apoptosis are, respectively, illustrated via percentages and dot images.

### Quantitative detection of the reactive oxygen species

Intracellular reactive oxygen species (ROS) was measured using the DCFH-DA fluorescent probe (Beyotime Biotechnology) according to the manufacturer’s instructions. Briefly, the primary astrocytes were seeded in 96-well plates and processed for treatments with different reagents for 24 h. Next, the culture media were removed, and 10 μM DCFH-DA was added to the cultures and incubated at 37 °C for 30 min. After washing thrice in PBS, the fluorescence intensities were measured on a microplate reader with a 485-nm excitation wavelength and a 35-nm emission wavelength.

### BrdU incorporation and TUNEL assay

The BrdU incorporation and TUNEL assays were further performed as previously described [21]. Briefly, to measure the proliferation activity, the cultured astrocytes were incubated with 10 μM bromodeoxyuridine (BrdU, Sigma) for 30 min. After fixation with 4 % PFA, the cells were incubated with 2 N HCl for 40 min at 37 °C and subsequently neutralized with 0.1 M borate buffer (pH 8.5) three times. After blocking with 1 % BSA for 1 h, the cells were probed with FITC-conjugated anti-BrdU antibody (Millipore, 1:100). To detect apoptosis, terminal deoxynucleotidyl transferase-dUTP nick end labeling (TUNEL) was performed with a commercial kit (Roche). Immunostaining fluorescence was observed under a laser scanning confocal microscope (LSCM, FV1000, Olympus, Japan).

### Immunohistochemistry and double immunofluorescence

For immunohistochemistry, the animals were perfused transcardially with 4 % paraformaldehyde fixation and sectioned for cryostat analyses. The midbrain sections containing the nigra were incubated in the following primary antibodies: mouse anti-TH (Sigma, 1:3000), rabbit anti-iba1 (Wako, 1:3000), mouse anti-GFAP (Millipore, 1:4000), rabbit anti-Gas1 (Santa Cruz, 1:100), rabbit anti-cleaved caspase-3 (Cell Signaling Technology, 1:500), and mouse anti-Ki-67 (Cell Signaling Technology, 1:700) in 0.01 M PBS containing 1 % bovine serum albumin and 0.1 % Triton X-100 for 24 h at 4 °C. After washing, the sections were incubated with the corresponding Alexa Fluo594-conjugated donkey anti-mouse and Alexa Fluo488-conjugated anti-rabbit IgGs for 2 h at room temperature. Immunostaining for the cultured astrocytes was also performed with a similar protocol. The nigral sections and cultured cells were observed, and the images of interest were captured under LSCM. For the quantitative data collection, the numbers of immunopositive cells were counted in every six sections (30-μm thickness) of the substantia nigra or six representative unit areas of cell cultures and averaged by two blinded observers.

### Real-time PCR

The total RNA was isolated from the primary astrocytes with Trizol reagent (Invitrogen) according to the manufacturer’s instructions. After reverse-transcription, the real-time PCR was performed using SYBR Green PCR Master Mix (TaKaRa). The primers for the rat Gas1 and GAPDH were as follows (forward and reverse): Gas1, 5′-GGATGTTGAGCGAATTAGGTT-3′ and 5′-CAGGAATGAATCGGTAAAGG-3′; GAPDH, 5′-ACAGCAACAGGGTGGTGGAC-3′ and 5′-TTTGAGGGTGCAGCGAACTT-3′.

### Western blot

The protein extraction and western blot were performed as described previously [20]. Fresh brain tissue or cultured cells were collected, and cell lysates were prepared in the lysis buffer (Beyotime Biotechnology). Next, after separation in sodium dodecyl sulfate polyacrylamide gel electrophoresis (SDS-PAGE) gels (10 or 12 %), the protein samples were transferred to PVDF membranes (Millipore). The membranes were blocked with 5 % nonfat milk/TBST (0.1 % Tween-20, TBS) for 1 h and incubated with the following antibodies: rabbit anti-Gas1 (Santa Cruz), mouse anti-TH (Sigma), mouse anti-GFAP (Millipore), rabbit anti-cleaved caspase-3 (Cell Signaling Technology), rabbit anti-iNOS (Abcam), mouse anti-p-NFkB-p65 (Sigma), rabbit anti-Bax (BBIAB, Sangon Biotech), rabbit anti-Bcl-2 (Bioworld), and rabbit anti-β-actin (Sigma) at 4 °C overnight, and β-actin was used as the loading control for the immunoblotting. After incubation with the appropriate HRP-conjugated secondary antibodies at room temperature for 1 h, the immunoblot bands were detected using an advanced ECL detection kit (Millipore). The immunoblots were digitally acquired using the Bio-Rad System. The quantitative data analyses of the immunoblots were performed and are shown in the density ratios compared with those of the control groups.

### Statistical data analysis

All quantitative data were collected from at least three animal samples or three independent experiments and are presented as the means ± the S.E.Ms. The statistically significant differences between groups or treatments were determined by one-way analysis of variation (ANOVA) with Student Newman-Keuls post hoc analyses. The level of significance was set at *P* < 0.05 for all analyses.

## Results

### Cellular localization and Gas1 up-regulation in the nigral astrocyte LPS rat model and MPTP mouse model

**Fig. 1:**
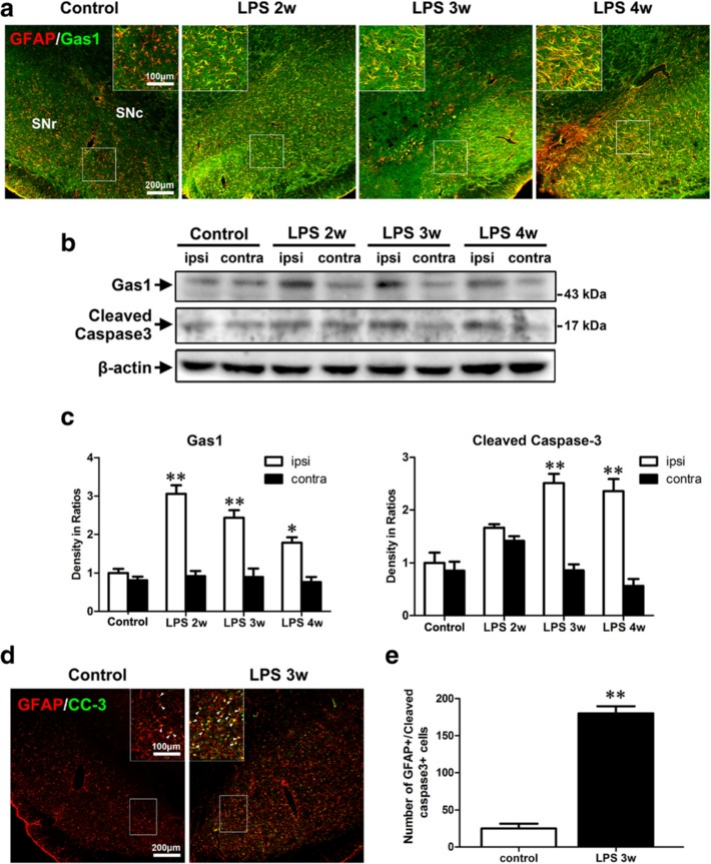
Induction of Gas1 expression and cleaved caspase-3 (CC-3) in the nigral astrocytes of LPS rat model. **a** Double labeling shows Gas1/GFAP-positive astrocytes in the substantia nigra of control and LPS group and magnified in inserts. **b** Immunoblots of Gas1 and CC-3 in the ventral midbrains of control and LPS-2-, 3-, and 4-week groups. **c** Comparison of Gas1 and CC-3 levels among above groups (density in ratios of interested groups compared with control). **d** GFAP/CC-3 double labeling in the nigra of control and LPS group. **e** Comparison of GFAP/CC-3-positive astrocytes between control and LPS group. ANOVA, *P < 0.05, **P < 0.01 vs control (mean ± S.E.M., *n* = 3)

**Fig. 2:**
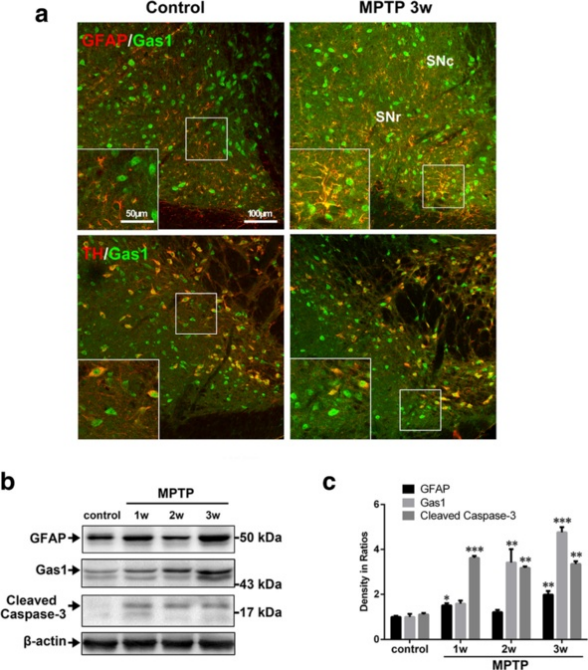
Expression of Gas1 and cleaved caspase-3 (CC-3) in the nigral astrocytes and neurons of MPTP mouse model. **a** Double labeling shows Gas1/GFAP-positive astrocytes and Gas1/TH-positive neurons in the substantia nigra of control and MPTP group and magnified in inserts. **b** Immunoblots of GFAP, Gas1, and CC-3 in the ventral midbrains of control and MPTP model. **c** Comparison of GFAP, Gas1, and CC-3 expression levels among control and MPTP group. ANOVA, *P < 0.05, **P < 0.01 vs control (mean ± S.E.M., *n* = 3)

The reactive astrocytes in the substantia nigra of the PD model were characterized with morphological changes in their enhanced proliferation and expression of intermediate filament GFAP [3]. The Gas1 expression profile of the reactive astrocytes was first examined in the nigra of the LPS rat model and MPTP mouse model, both of which exhibited significant inflammatory responses. At 2, 3, and 4 weeks after the stereotactic injection of LPS in the nigra, the midbrain sections were doubly labeled with Gas1/GFAP immunofluorescence. The Gas1 in the GFAP-positive astrocytes was up-regulated and accompanied by remarkable astrogliosis in the substantia nigra. Due to the host restriction of the antibodies for double immunofluorescence, Gas1 and iba1 (a microglia marker) were not doubly labeled. Gas1/GFAP-double labeled astrocytes almost occupied all the shape of glial cells, which indicated that microglia might not express or express very lowly levels of Gas1 in the nigra (Fig. 1a, Additional file 1: Figure S1A). The positive expression of Gas1 was then confirmed in the reactive nigral astrocytes of the MPTP model (Fig. 2a). Additionally, Gas1-immunoreactivity was also observed in numerous nigral neurons indicated by Gas1/TH double immunofluorescence (Fig. 2a, Additional file 2: Figure S1B).

The tissue lysates dissected from the ventral midbrain of the LPS rat model and the MPTP mouse model were analyzed via western blotting. The significant increases in the Gas1 expression level were detected after the LPS and MPTP insults (Figs. 1b, c and 2b, c), which were basically consistent with double labeling. Regarding specifically confirming the Gas1 antibody used, the cultured SH-SY5Y cells were applied as a positive immunoblot control (Additional file 1: Figure S2A, B) and had previously been reported to express Gas1 protein under a serum-deprived state [15]. Moreover, the appearance and molecular weight of the detected Gas1 immunoblot bands were also clearly consistent with previous literature [22–24].

### Apoptotic appearance of astrocytes accompanied with Gas1 up-regulation in the injured nigra of the LPS and MPTP models

Cleaved caspase-3-positive astrocytes were numerously observed in the substantia nigra after the LPS insult, but few were detected in the contralateral side at the same time-point (Fig. 1d, e). Consistently, western blotting revealed the significant expression of cleaved caspase-3 in the substantia nigra of the LPS model rats and the MPTP model mice (Figs. 1b–e and 2b, c). Thus, these findings indicated that the up-regulated Gas1 expression was accompanied by an obvious activation of the caspase-3-dependent pathway in the reactive astrocytes, which suggested the enrolment of increased Gas1 signaling in the apoptosis of these activated astrocytes in the injured substantia nigra under the LPS and MPTP insults.

At the same time, the nigral neurons in both the LPS rat model and MPTP mouse model were confirmed and the neuronal degeneration or injury of the nigral TH-positive neurons were observed as reported in our previous studies [20, 25]. Indeed, Gas1 expression was clearly observed in both the nigral TH-positive dopaminergic neurons and the GFAP-positive reactive astrocytes, and the possible implication of Gas1 up-regulation in the mediation of neuronal apoptosis has been evidenced in Gas1 functional studies in SH-SY5Y cells under serum-deprived states and in also in the hippocampus [15, 16].

### Dynamic changes in Gas1 expression in primary astrocytes in relation to cell density

**Fig. 3:**
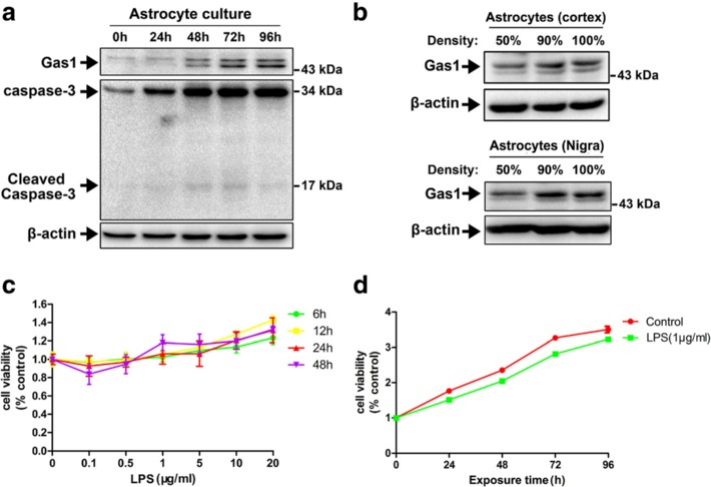
Expression of Gas1 in primary astrocytes and influence of LPS on cell viability of astrocytes. **a** Immunoblots of Gas1 expression and cleaved caspase-3 in the astrocytes with increasing culture times of 24, 48, 72, and 96 h. **b** Immunoblots of Gas1 expression levels in the astrocytes with increasing cell density from the cerebral cortex and substantia nigra. **c** Cell viability of the astrocytes with LPS insult at increasing concentrations of 0.1, 0.5, 1, 5, 10, and 20 μg/ml. **d** Cell viability of the astrocytes under control and LPS (1 μg/ml) insult with increasing times of 24, 48, 72, and 96 h

In the cell culture, the influence of cell density on Gas1 expression in the primary astrocytes was analyzed because Gas1 may be involved in the contact inhibition of cell proliferation. Primary astrocytes were plated at a low cell density in DMEM with 10 % FCS and cultured for 0, 24, 48, 72, and 96 h until they achieved confluences of up to 100 %. The levels of Gas1 and cleaved caspase-3 increased gradually in the astrocytes at higher confluences (Fig. 3a). To confirm whether this effect was consistent for astrocytes from different brain regions, astrocytes isolated from the cerebral cortex and nigra were plated at low (50 %) and high (90 and 100 %) densities in DMEM with 10 % FCS and lysed at the same time. The levels of Gas1 expression increased in the astrocytes from both brain regions at the higher cell densities in similar patterns (Fig. 3b).

Cell viability was then measured in the astrocytes that were insulted with LPS at different concentrations and different treatment periods of 0–96 h. A CCK-8 assay revealed that LPS alone had no significant effect on the cell viabilities of the astrocytes after treatments with various LPS concentrations (0–20 μg/ml) at the time-points of 6, 12, 24, and 48 h. There were also no detectable changes in cell viability in the LPS group compared with the controls at the time-points of 0 to 96 h in the primary astrocytes (Fig. 3c, d).

### Gas1 up-regulation, increased ROS generation, and decreased cell viability of the astrocytes induced by exposure to LPS, IFNγ, and TNFα

**Fig. 4:**
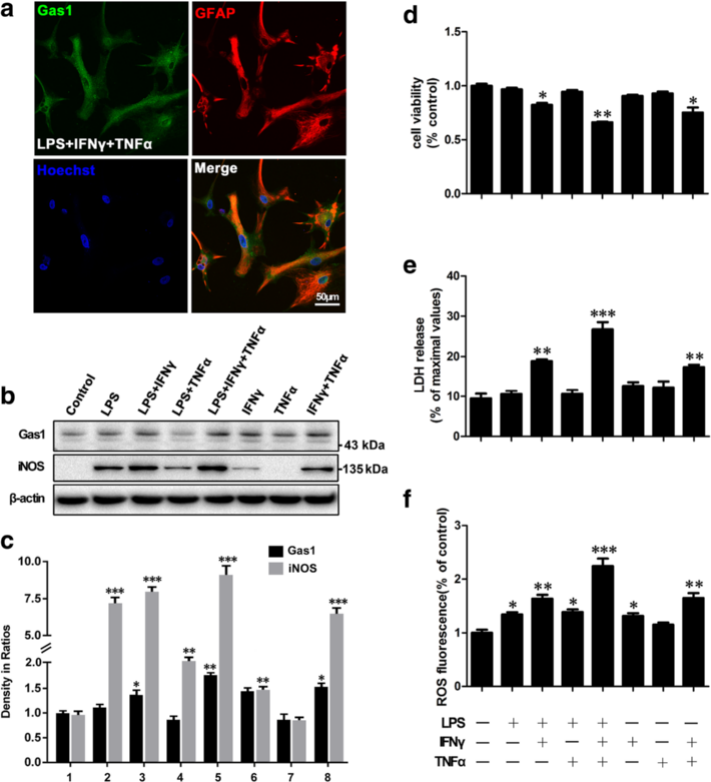
Influence of LPS, IFNγ, TNFα, and their combinations on expression of Gas1 and iNOS, cell viability, and cell death, and generation of reactive oxygen species (ROS) in cell culture of astrocytes. **a** Gas1-immunoreactivity in astrocytes after treatment with LPS, IFNγ, and TNFα. **b** Immunoblots of Gas1 and iNOS in the astrocytes with distinct treatments at 24 h. **c** Comparison of Gas1 and iNOS levels among above groups. **d** Cell viability determined by CCK-8 assay. **e** Cell death determined by LDH release assay. **f** ROS generation assay in above distinct groups of astrocytes. ANOVA, *P < 0.05, **P < 0.01, ***P < 0.001 vs control (mean ± S.E.M., *n* = 3)

After excluding the direct cytotoxicity of the LPS exposure of the primary astrocytes, we further examined the effects of combinations of LPS and its induced products IFNγ and TNFα from microglial cells or lymphocytes in neuroinflammation events [26]. To demonstrate the influence of Gas1 changes on iNOS, ROS, cell viability, and cell death, LPS, IFNγ, TNFα, and their combinations were applied to cultured astrocytes (Fig. 4). The results revealed that no significant changes were observed in Gas1 expression, iNOS, ROS, cell viability, or death in the controls. In the LPS + IFNγ + TNFα, LPS + IFNγ, and IFNγ + TNFα groups, the up-regulation of Gas1 expression was significant and accompanied by an enhanced iNOS level, increased ROS generation, decreased viability, and increased death of the astrocytes (Fig. 4a–f). In the LPS, LPS + TNFα, and IFNγ groups, Gas1 expression remained constant along with unchanged cell viability and death, while increases in iNOS and ROS levels were still observed. Unexpectedly, exposure to TNFα singly did not result in obvious effects on Gas1, iNOS, ROS, cell viability, or astrocyte death compared with the controls.

Furthermore, similar changes in viability were observed in the astrocytes that were isolated from different brain regions, i.e., the cerebral cortex, nigra, and striatum (Additional file 2: Figure S2C, D). The data collectively indicated that the up-regulation of the Gas1 expression level was consistent with the decreased cell viability and increased cell death of the primary astrocytes, and it partially conformed to the increases in the levels of iNOS and ROS generation. Thus, these findings also suggested that the increases in iNOS and ROS generation might have possibly contributed to the Gas1-induced apoptosis of the reactive astrocytes following exposure to certain inflammatory cytokines, most obviously after the stimulation with the combination of LPS, IFNγ, and TNFα.

### Decreased viability of astrocytes induced by LPS, IFNγ, and TNFα via influences on cell apoptosis and cell proliferation

**Fig. 5:**
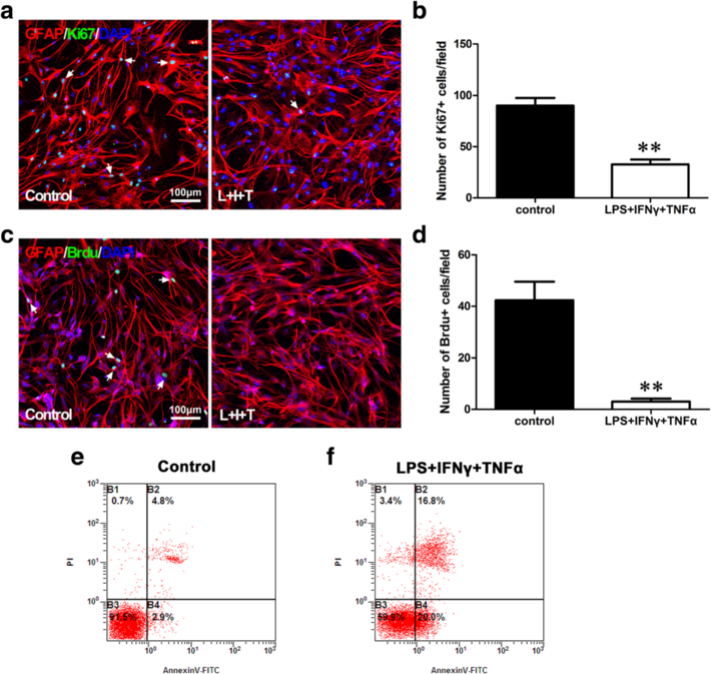
Influence of LPS, IFNγ, and TNFα on cell proliferation, survival, and apoptosis of primary astrocytes. **a** GFAP/Ki-67 double labeling in astrocytes of control and LIT (LPS + IFNγ + TNFα) group. **b** Comparison of Ki-67-positive cells per unit field between control and LIT group. **c** GFAP/BrdU double labeling in astrocytes of control and LIT group. **d** Comparison of BrdU-positive cells per unit field between control and LIT group. **e**, **f** Flow cytometry shows survival, necrosis and apoptosis of astrocytes of control and LIT group by Annexin V and PI staining. *B1* necrotic cells; *B2* late apoptotic cells; *B3* viable cells; *B4* early apoptosis. ANOVA, **P < 0.01 vs control (mean ± S.E.M., *n* = 3)

To examine the possible reasons for the viability decreases of the primary astrocytes following exposure to LPS, IFNγ, and TNFα, the cell proliferation and apoptosis of the astrocytes were evaluated via the detection of Ki-67 and BrdU. Ki-67 is a common proliferative marker of phases G1, S, G2, and M, and BrdU is a specific marker of incorporation in the S phase of cell division. Double labeling revealed Ki-67- and BrdU-positive cells among the astrocytes of the controls but few among the astrocytes following exposure to the LPS, IFNγ, and TNFα treatment (Fig. 5a, c). Cell counts indicated that the numbers of Ki-67- and BrdU-positive astrocytes were decreased among the astrocytes treated with LPS, IFNγ and TNFα compared with the controls (Fig. 5b, d).

A flow cytometry assay was performed by Annexin V-PI staining to indicate cell survival, apoptosis, and necrosis among the astrocytes that were with the combination of LPS, IFNγ, and TNFα. The results revealed that the apoptotic proportions of astrocytes in both the early and later stages of apoptosis were increased in the groups exposed to LPS, IFNγ, and TNFα treatment compared with the control (Fig. 5e, f). The surviving proportions of astrocytes subjected to LPS, IFNγ, and TNFα treatment also decreased accordingly. The data thus suggested that the decrease in the cell viability of the astrocytes following the treatment with LPS, IFNγ, and TNFα might have been attributable to both the inhibition of cell proliferation and apoptosis.

### Knock-down of Gas1 rescued astrocyte viability via the inhibition of Bax expression, the activation of cleaved caspase-3, and cell apoptosis

**Fig. 6:**
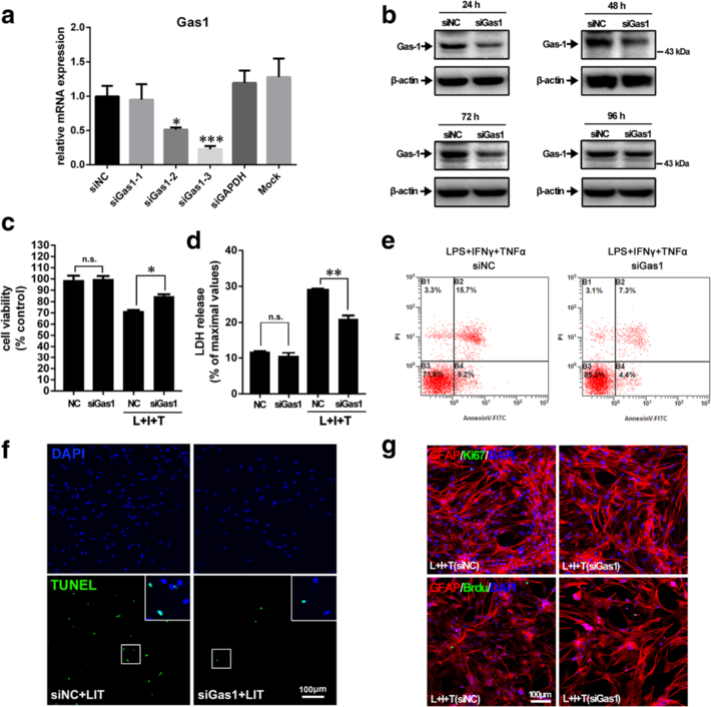
Effect of siRNA knockdown of Gas1 expression on survival, apoptosis, and proliferation of the primary astrocytes. **a** siRNA knockdown efficiency of three small interfering RNA specific towards Gas1 (siGas1) determined by real time qPCR. *P < 0.05, ***P < 0.001 vs control (mean ± S.E.M., *n* = 3). **b** Effective working time of siGas1 measured by immunoblot. **c**, **d** Influence of down-regulated Gas1 on cell viability or necrotic death of astrocytes of control and treated with LPS, IFNγ and TNFα by CCK-8 and LDH assay. ANOVA, *P < 0.05, **P < 0.01, ***P < 0.001 vs control, *n.s.* no significance (mean ± S.E.M., *n* = 3). **e** Influence of down-regulated Gas1 on survival, apoptosis, and necrosis of primary astrocytes by flow cytometry. **f** Influence of Gas1 siRNA on apoptosis of cultured astrocytes treated with LPS, IFNγ, and TNFα by TUNEL staining. **g** Influence of Gas1 siRNA on proliferation state of astrocytes treated with LPS, IFNγ, and TNFα by Ki-67/BrdU double labeling

To observe the implications of the Gas1 signal in the inhibition of the proliferation and apoptosis of the reactive astrocytes treated with LPS, IFNγ, and TNFα, Gas1 knockdown and biological effect experiments were performed. Cultured astrocytes were transfected with three designed Gas1 siRNAs, and the total RNA was isolated for real-time PCR analysis at 48 h. The results revealed that the siGas1-3 effectively down-regulated Gas1 expression in the astrocytes compared with the scrambled siRNA control (Fig. 6a). siGas1-3 was selected for the biological effect analysis of Gas1 knockdown. Western blotting confirmed that the knockdown or decrease in Gas1 protein expression was sustained for at least 72 h (Fig. 6b).

To study the influence of Gas1 gene knockdown on cell apoptosis among the reactive astrocytes caused by exposure to LPS, IFNγ, and TNFα, a CCK-8 assay, LDH release, flow cytometry assay, TUNEL staining, and GAFP/Ki-67 and GFAP/BrdU double-labeling were performed. The decrease in cell viability and the increase in LDH release were significantly rescued when Gas1 expression was down-regulated in advance by the administration of the siRNA (Fig. 6c, d). Flow cytometry and TUNEL assays further revealed that the apoptosis of the astrocytes caused by the LPS, IFNγ and TNFα treatment could be inhibited by the knockdown of Gas1 expression (Fig. 6e, f). Intriguingly, however, the knockdown of Gas1 did not obviously change the numbers of Ki-67- or BrdU-positive astrocytes treated with LPS, IFNγ, and TNFα (Fig. 6g).

**Fig. 7:**
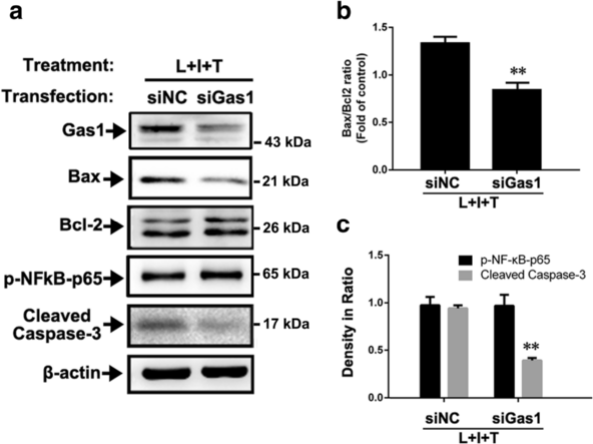
Influence of siRNA on Gas1 knockdown, Bax, Bcl-2, p-NFkB-p65, and cleaved caspase-3 levels in primary astrocytes treated with LPS, IFNγ, and TNFα. **a** Immunoblots of Gas1, Bax, Bcl-2, p-NFkB-p65, and cleaved caspase-3 in siNC and siGas1 group. **b** Comparison of Bax/Bcl-2 ratios between siNC and siGas1 group. **c** Comparison of p-NFkB-p65 and cleaved-caspase-3 levels between siNC and siGas1 group. ANOVA, **P < 0.01 vs siNC group

**Fig. 8:**
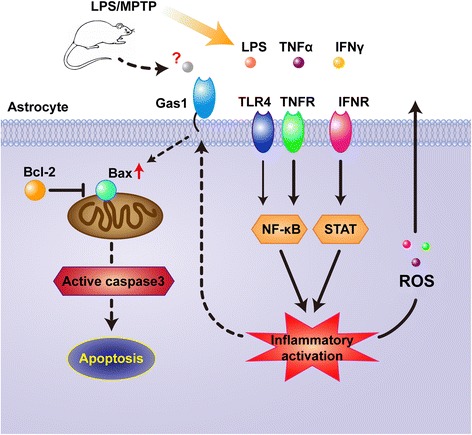
A schematic showing of Gas1 functional ways in mediating apoptosis of reactive astrocytes, in which up-regulation of inducible Gas1 signaling might be resulted from inflammatory NFkB activation, and then possibly participate in increasing reactive oxygen species (ROS) or oxidative stress, enhancing Bax/Bcl-2 ratios, and finally triggering caspase-3 activation-related apoptosis of reactive astrocytes in LPS and MPTP models or inflammatory events

Finally, a potential mechanism underlying the Gas1-induced apoptosis was observed in reactive astrocytes following inflammatory treatment with LPS, IFNγ, and TNFα. The knock-down effects of Gas1 siRNA on Gas1 expression were examined in relation to changes in apoptosis-related signaling molecules, such as Bax, Bcl-2, and cleaved caspase-3, in the astrocytes. Western blotting revealed that Gas1 siRNA effectively decreased the Bax expression and the cleaved caspase-3 level, but it did not significantly alter the Bcl-2 or p-NFkB-p65 levels in the astrocytes that were treated with LPS, IFNγ and TNFα (Fig. 7a–c). These data together suggested that the up-regulated Gas1 signal might have induced apoptosis among the reactive astrocytes by increasing the Bax/Bcl-2 ratio, enhancing ROS generation, and triggering the activation of the caspase-3-dependent signaling pathway during neuroinflammatory events (Fig. 8).

## Discussion

In this study, we investigated the expression profile and dynamic changes in Gas1 in the brains of LPS and MPTP models and presented the new finding that Gas1 expression was dramatically induced in reactive astrocytes in the substantia nigra. The knockdown of Gas1 gene expression in the primary astrocytes further confirmed that the induction of Gas1 expression in these activated astrocytes accelerated the elimination of these cells via Bax/caspase-dependent apoptosis. The data from this study together suggest a pivotal regulatory role of Gas1 in the apoptosis of reactive astrocytes or astrogliosis in the injured substantia nigra regions of PD models.

Reactive astrogliosis is well established to be actively involved in the entire pathological process of the PD condition. However, whether astrogliosis plays a beneficial or harmful role remains to be established [27, 28]. Although neurotrophic factors, such as glial-derived neurotrophic factor (GDNF), basic fibroblast growth factor, and brain-derived neurotrophic factor (BDNF), have been identified in these activated astrocytes [25, 29], a line of growing evidence has demonstrated that LPS or inflammatory factors also trigger astrocytes to release the pro-inflammatory cytokines NO and ROS, which are detrimental to the neighboring neurons [5, 30, 31]. Genomic analysis of an LPS mouse model revealed that the activated astrocytes exhibit a detrimental phenotype [32]. Consistently, we observed significant increases in the pro-inflammatory factors iNOS and ROS in the activated astrocytes following stimulation with LPS in combination with other cytokines. Thus, the identification of new regulators that control inflammatory astrogliosis and the prevention of the progression of PD by the elimination of these activated astrocytes with detrimental properties may be essential.

Gas1 is a pleiotropic protein that can inhibit cell growth and induce apoptosis in cancer cells and actively participates in mammalian embryonic development [33]. Gas1 expression has been widely found in the CNSs of adult rodents, and Gas1 is abundantly localized in both astrocytes and neurons [19]. While the biological implications of Gas1 in the mediation of neuronal apoptosis have been demonstrated in functional studies of Gas1 in SH-SY5Y cells and the hippocampus [15, 16], the potential role of Gas1 in astrocytes remains unclear. An interesting finding of this study is that Gas1 up-regulation was induced in reactive astrocytes and accompanied by apoptosis in the injured nigras of the LPS rat model and the MPTP mouse model of PD. Additionally, we also identified the gradual up-regulation of Gas1 and GFAP expression levels in the midbrain with the increasing ages of the rats (data not shown). The increase in reactive astrocytes is one of the most identified and accepted biomarkers of brain aging [34] and might partially explain the PD susceptibility of older populations. In astrocyte cell cultures, Gas1 expression is also remarkably increased following the administration of the combination of LPS, IFNγ, and TNFα. The expression trend of Gas1 was partially consistent with the iNOS and ROS levels, which suggests that the increases in iNOS and ROS might possibly contribute to the Gas1-induced apoptosis of reactive astrocytes following exposure to inflammatory cytokines, which was most obvious following stimulation with the combination of LPS, IFNγ, and TNFα.

Accumulating evidence has demonstrated the over-activation-induced cell death of astrocytes in vivo and in vitro. LPS-induced systemic inflammation can increase iNOS and the activations of both astrocytes and microglial cells in the hippocampus and midbrain, and these activations are paralleled by the time-dependent apoptosis of astrocytes and microglia [35]. The apoptosis of astrocytes has also been observed in the brains of Alzheimer’s disease [7] and HIV encephalitis [8] patients. Nitric oxide (NO) produced in vitro by activated astrocytes has been found to not only damage surrounding neurons but also induce the apoptosis of astrocytes in a caspase-dependent manner [10, 36]. Our results indicated that the stereotactic injection of LPS also induced significant apoptosis among activated astrocytes in the injured nigras. However, LPS in vitro alone did not exhibit an effect on the viability of primary astrocytes at different concentrations and treatment periods. One possible explanation is that the relative lack of TLR4 in astrocytes mediated the LPS effect compared with the TLR4 signaling of the microglial cells [4, 37]. Astrocytes were recently reported to indirectly respond to LPS through the release of soluble mediators from the surrounding microglia [38], neurons, or other immune cells. Among these soluble mediators, TNFα and IFNγ are the two major pro-inflammatory factors, and the TNFα and IFNγ receptors are remarkably up-regulated in activated astrocytes [39, 40]. Consistently, LPS, TNFα, or IFNγ alone were unable to induce astrocyte death, but the combination of LPS, TNFα, and IFNγ decreased the viability of the primary astrocytes, and this decrease was paralleled by increased iNOS and ROS levels. Some authors have attributed the death of astrocytes to NO toxicity due to autocrine release, and the administration of N-monomethyl L-arginine, an inhibitor of iNOS, can partially block the cell death of activated astrocytes [10, 36, 41]. Additionally, the present study also provided evidence that the decrease in astrocyte viability might also be partially the result of the inhibition of proliferation caused by LPS, TNFα, or IFNγ stimulation.

Moreover, Gas1/TH double immunofluorescence staining was also performed in the LPS and MPTP animal models. Clearly, Gas1-immunoreactivity was also detected in the cytoplasm and nuclei of the nigral neurons, and nearly all TH-positive neurons, exhibited Gas1 positivity. We also confirmed the gradual elevation of Gas1 expression in the SH-SY5Y dopaminergic cells that was accompanied by apoptosis in a serum-deprived condition [15]. Gas1 knockdown increases the protective effect of GDNF against glutamate-induced injury in SH-SY5Y cells [23], and Gas1 has been reported to be induced and involved in the excitotoxic deaths of hippocampal neurons [16]. Thus, the data from the present study may also propose a critical question regarding the enrollment of the up-regulation of the Gas1 signal in the mediation of the apoptosis of dopaminergic neurons in the substantia nigra during the pathogenesis of PD.

Finally, we confirmed relationship between the Gas1 expression level and the cell viability of the activated astrocytes via a siRNA knock-down experiment. The results of this experiment indicated that the knock-down of Gas1 rescued the cell viability of the astrocytes via the inhibition of apoptosis. Gas1 is a well-known growth arrest protein that has been proven to block the transition of the G_0_ to the S phase in fibroblasts [42], glioblastomas [43], and breast cancer cells [44]. As a pleiotropic protein, Gas1 appears to exert multiple functional roles in distinct cell types. The intracellular signaling analysis of the present study revealed that the potential underlying mechanism of astrocyte apoptosis induced by LPS, TNFα, and IFNγ treatment was evidently related to the up-regulation of Bax, which is a pro-apoptotic protein that follows the activation of caspase-3. The knock-down of Gas1 was confirmed to decrease the Bax/Bcl-2 ratio, alleviate caspase-3 activation, and ultimately diminish the apoptosis of reactive astrocytes. This point is supported by the previous clue regarding Gas1-induced cell apoptosis due to the Bax/Bcl-2 ratio and caspase-3 activation in gastric cancer [14]. Nevertheless, this work should merit further investigations of the precise functions of Gas1 in the regulation of astrogliosis, astrocyte survival, and apoptosis to further understand the inflammatory responses and pathological progression of PD in human beings.

## Conclusions

This study identified Gas1 as a novel modulator in apoptosis in reactive astrocytes via in vivo and in vitro experiments. The up-regulation of Gas1 expression was inducible in reactive astrocytes in the injured nigras of LPS and MPTP models and was correlated with the apoptosis of these reactive astrocytes. While the up-regulation of Gas1 was able to induce apoptosis and the inhibition of the proliferation of astrocytes following exposure to LPS, IFNγ, and TNFα, the knockdown of Gas1 exhibited a rescuing effect on the astrocytes via modulations of the Bax/Bcl-2 ratio and cleaved caspase-3 activation and apoptosis. Taken together, these results indicate that the Gas1 signal may work as new regulator of cell survival and apoptosis of reactive astrocytes. The potential underlying mechanisms are illustrated schematically in Fig. 8. Hopefully, further studies of the functions of Gas1 related to the regulation of astrogliosis, astrocyte survival, and apoptosis will benefit the understanding of the inflammatory response and the establishment of a novel intervention against inflammatory injury in PD conditions.

## Abbreviations

BrdU, bromodeoxyuridine; Gas1, growth arrest specific 1; GFAP, glial fibrillary acidic protein; IFNγ, interferon γ; iNOS, inducible nitric oxide synthase; LDH, lactate dehydrogenase; LPS, lipopolysaccharide; MPTP, 1-methyl-4-phenyl-1,2,3,6-tetrahydropyridine; NFkB, nuclear factor kB; PCNA, proliferative cell nuclear antigen, PD, Parkinson’s disease; ROS, reactive oxygen species; TH, tyrosine hydroxylase; TNFα, tumor necrosis factor α

## Additional files

Additional file 1: Figure S1. Gas1/GFAP and Gas1/TH double labeling shows localization of Gas1 in both astrocytes and neurons in the substantia nigra of LPS and MPTP model by each fluorescence channel images. A-A”, Gas1 single, GFAP single, and Gas1/GFAP merged images in the nigral astrocytes of LPS rat model-3w; B-B”, Gas1 single, TH single, and Gas1/TH merged images in nigral dopaminergic neurons of MPTP mouse model-3w. (TIF 2545 kb) Additional file 2: Figure S2. (A) Western blotting verification of Gas1 expression (H-300 antibody) and cleaved caspase-3 in SH-SY5Y cells under serum-deprived state with increasing culture times; (B) Comparison of Gas1 and cleaved caspase-3 levels in SH-SY5Y cells among distinct culture time-points of 0, 24, 48, 72 and 96 h; (C, D) Influence of LPS, IFNγ and TNFα on cell viability of primary astrocytes isolated from the substantia nigra and striatum. ANOVA, *P < 0.05, **P < 0.01, ***P < 0.001 *vs* control (mean ± S.E.M., n = 3). (TIF 2777 kb)
