# Supplementary figures and images for: Gas1 up-regulation is inducible and contributes to cell apoptosis in reactive astrocytes in the substantia nigra of LPS and MPTP models
Source: J Neuroinflammation. 2016 Jul 8;13:180. doi: 10.1186/s12974-016-0643-2 (PMC4938987; doi:10.1186/s12974-016-0643-2)

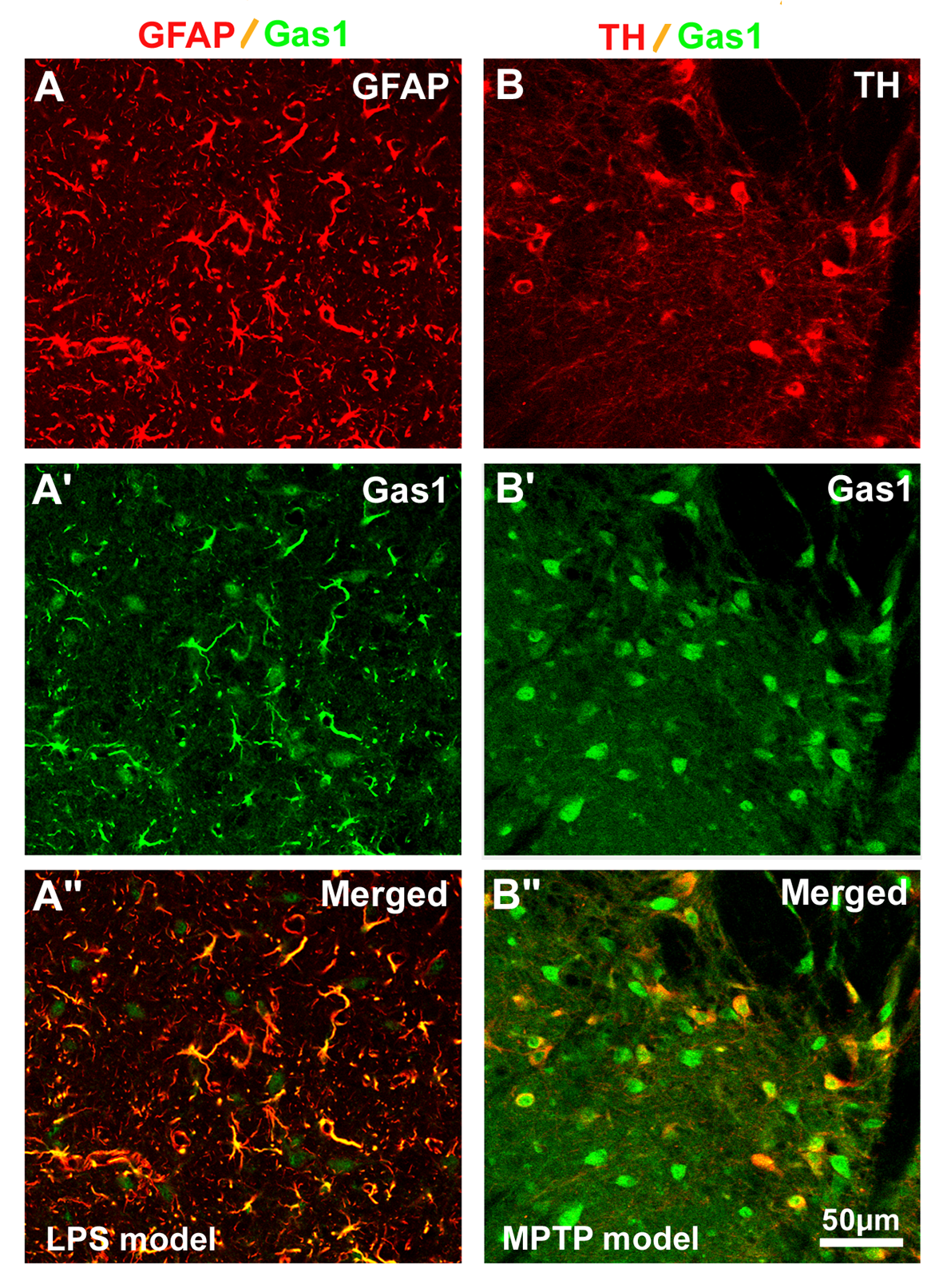

Supplement: Additional file 1: Figure S1. — Gas1/GFAP and Gas1/TH double labeling shows localization of Gas1 in both astrocytes and neurons in the substantia nigra of LPS and MPTP model by each fluorescence channel images. A-A”, Gas1 single, GFAP single, and Gas1/GFAP merged images in the nigral astrocytes of LPS rat model-3w; B-B”, Gas1 single, TH single, and Gas1/TH merged images in nigral dopaminergic neurons of MPTP mouse model-3w. (TIF 2545 kb) [file 12974_2016_643_MOESM1_ESM.tif]

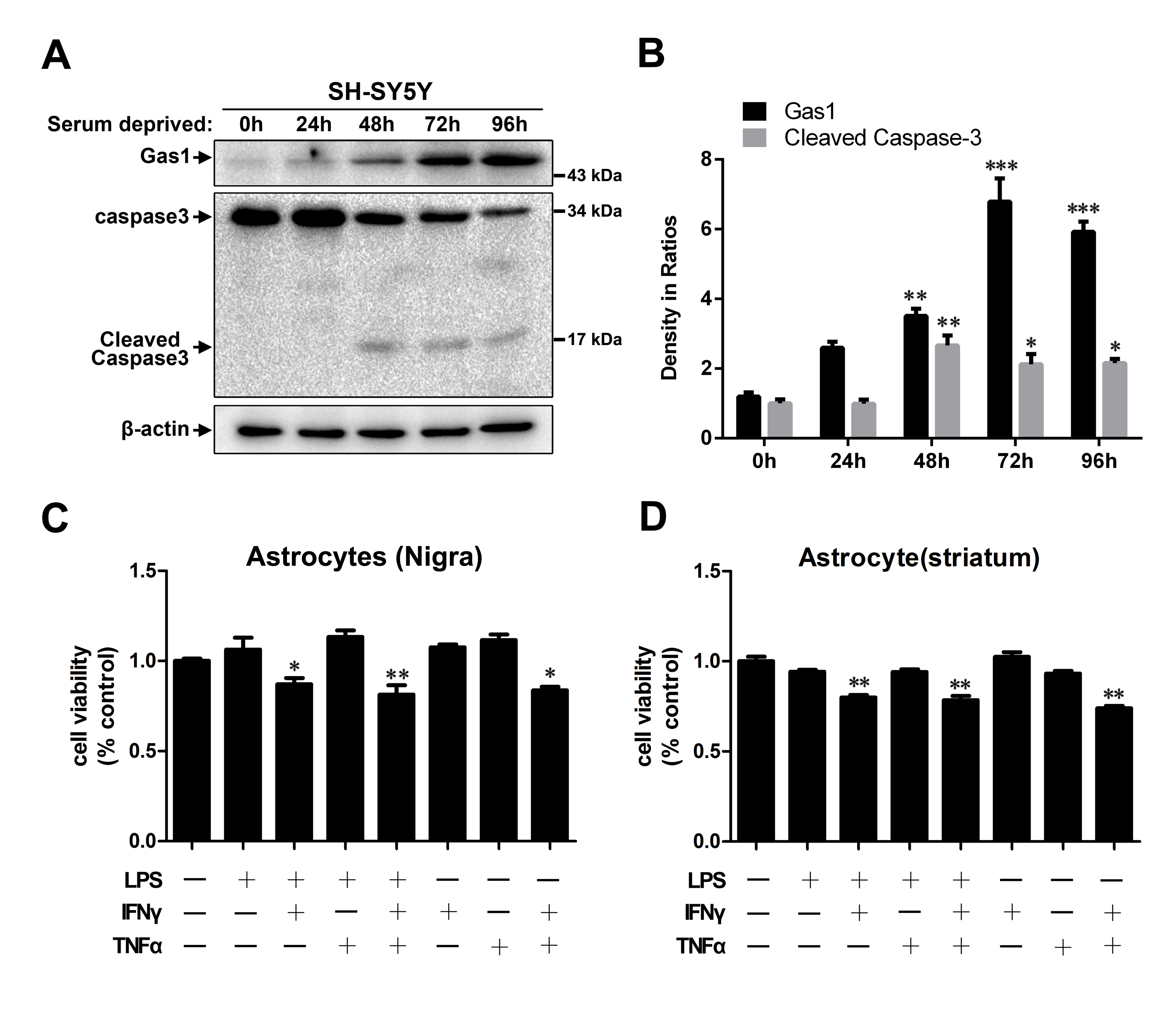

Supplement: Additional file 2: Figure S2. — (A) Western blotting verification of Gas1 expression (H-300 antibody) and cleaved caspase-3 in SH-SY5Y cells under serum-deprived state with increasing culture times; (B) Comparison of Gas1 and cleaved caspase-3 levels in SH-SY5Y cells among distinct culture time-points of 0, 24, 48, 72 and 96 h; (C, D) Influence of LPS, IFNγ and TNFα on cell viability of primary astrocytes isolated from the substantia nigra and striatum. ANOVA, *P < 0.05, **P < 0.01, ***P < 0.001 vs control (mean ± S.E.M., n = 3). (TIF 2777 kb) [file 12974_2016_643_MOESM2_ESM.tif]
